# Importance of Vaccination Against COVID-19 in a Patient With End-Stage Rheumatoid Arthritis-Associated Interstitial Lung Disease (RA-ILD)

**DOI:** 10.7759/cureus.63810

**Published:** 2024-07-04

**Authors:** Bella Garg, Amirmohsen Arbabi, Purnell A Kirkland, Paryus Patel

**Affiliations:** 1 Internal Medicine/Rheumatology, Centinela Hospital Medical Center, Los Angeles, USA; 2 Internal Medicine, Centinela Hospital Medical Center, Los Angeles, USA; 3 Rheumatology, Centinela Hospital Medical Center, Los Angeles, USA; 4 Internal Medicine/Pulmonology, Centinela Hospital Medical Center, Los Angeles, USA

**Keywords:** vaccination, covid-19, pulmonary fibrosis, interstitial lung disease, rheumatoid arthritis

## Abstract

Rheumatoid arthritis (RA) is a chronic autoimmune disease characterized by inflammatory polyarthritis and extra-articular involvement. Extraarticular manifestations of RA can include involvement of the skin, eye, heart, lungs, and others. RA is associated with a broad spectrum of pleuropulmonary involvement, with interstitial lung disease (ILD) and pleural disease being the most common. COVID-19 infection cross-talks with RA at various stages of pathogenesis. The clinical course and outcome of COVID-19 infection in RA patients may be ameliorated due to various reasons including anti-rheumatic drugs; however, COVID-19 vaccination provides additional protection to high-risk patients compared to non-vaccinated patients. Here, we present a case of end-stage RA-associated ILD who presented with the chief complaint of shortness of breath and tested positive for COVID-19. She was a lung transplant candidate on long-term antifibrotic medication nintedanib for interstitial fibrosis. The patient survived the initial acute hypoxemic respiratory failure, which might be attributed to being fully vaccinated for COVID-19.

## Introduction

Rheumatoid arthritis (RA) is a chronic autoimmune multisystemic inflammatory disease that involves multiple joints bilaterally. Extra-articular manifestations of RA occur in about 40% of patients at any time during the course of the disease, including in the early stages [1]. One of the most frequent extra-articular manifestations of RA is pleuropulmonary involvement, which encompasses interstitial lung disease (ILD), pleural disease, airway disease, rheumatoid nodules, pulmonary hypertension and drug-induced toxicity [2]. Patients with chronic autoimmune disorders like RA are at higher risk of infection because of the autoimmune condition itself and the treatment with immunosuppressive medications [3]. The prevalence of COVID-19 infection may not be significantly higher in RA patients, but these patients, notably those with extraarticular complications such as ILD, were at higher risk of severe COVID-19 infection than the general population especially early in the pandemic [4,5]. The in-hospital mortality rate in COVID-19 patients with RA is significantly higher compared to COVID-19 patients without RA [6]. However, full vaccination history for COVID-19 is found to be associated with a reduced number of ICU admissions, non-invasive ventilation, and length of hospital stays, thus ultimately reducing the mortality rate in critically ill COVID-19 patients [7-9]. Here, we discuss a patient with RA-associated ILD (RA-ILD) who presented to the emergency department (ED) with acute on chronic hypoxic respiratory failure and tested positive for COVID-19. She was on long-term antifibrotic medication nintedanib for interstitial fibrosis and was a lung transplant candidate. This case report aims to provide awareness regarding the importance of COVID-19 vaccination in the general population, especially in patients with RA-associated ILD.

## Case presentation

This case highlights a 60-year-old female, with a history of rheumatoid arthritis, RA-ILD, pulmonary fibrosis on nintedanib, diabetes mellitus, and hypertension, who presented to the ED with the chief complaint of shortness of breath and cough for the past two weeks. Per emergency medical services (EMS), her oxygen saturation was 70% on room air, which increased to 95% on 4 liters per minute (L/min) oxygen through a nasal cannula (NC). Initial vital signs on admission revealed a temperature of 37.1 °C, a blood pressure of 130/90 mmHg, a pulse rate of 105/minute, and a respiratory rate of 18/minute with an oxygen saturation of 96% on 6 L/min through an NC. Additionally, the patient complained of mild fever, yellow sputum production, generalized body aches, and fatigue. She mentioned that she had received four doses of Pfizer COVID-19 vaccines (New York, New York, US) since the beginning of the pandemic. She also reported being diagnosed with RA-ILD/pulmonary fibrosis five years ago, for which she has been taking nintedanib 150 mg every 12 hours orally. Significant findings on physical examination included decreased bibasilar breath sounds, inspiratory crackles/rales, expiratory wheezes, and mild to moderate respiratory distress.

**Figure 1:**
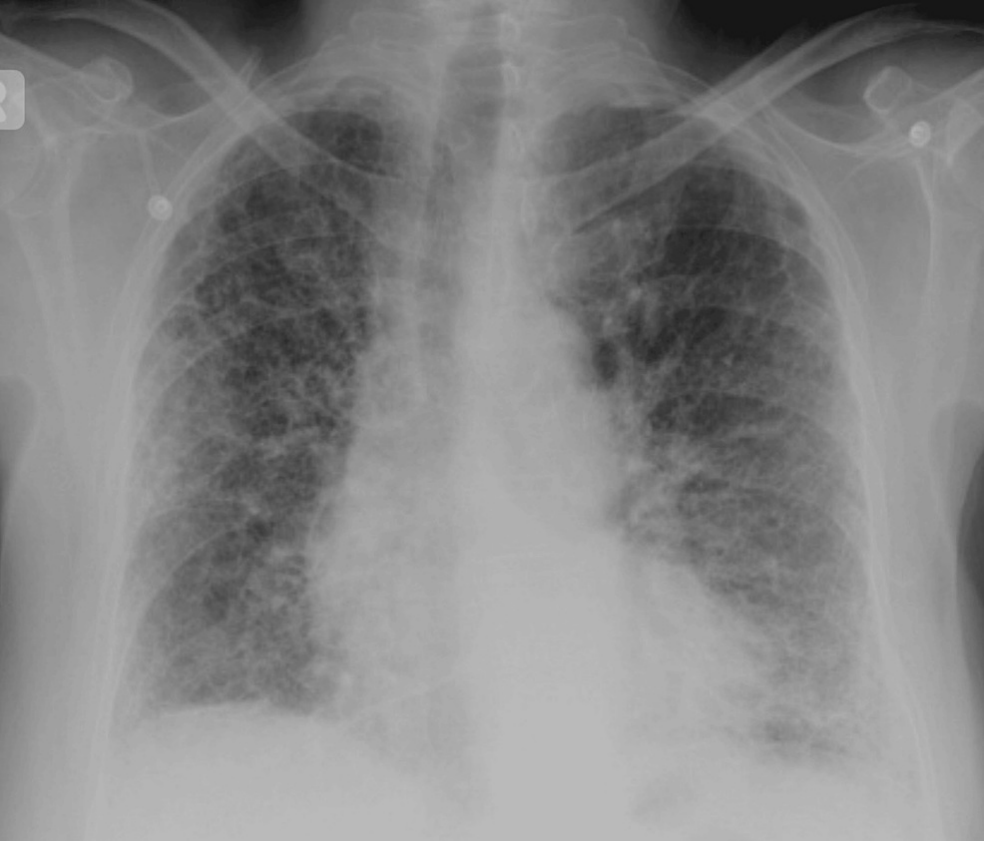
The chest X-ray showed bilateral pulmonary opacities suggestive of COVID-19 pneumonia with an underlying pulmonary fibrosis

The initial laboratory evaluation displayed the presence of a normal white blood cell (WBC) count at 5.5 x 1000/µL; macrocytosis with an MCV of 105 and a hemoglobin level of 14 g/dL; normal platelet count of 170 x 1000/µL; elevated lactic level acid of 4.7 mmol/L; normal procalcitonin level of 0.09 ng/mL; elevated D-dimer of 2.17 mg/L; normal ferritin of 251 ng/mL; low albumin of 2.7 g/dL; and hemoglobin A1C of 7.1%. The kidney function tests presented with a creatinine level of 0.7 mg/dL, a blood urea nitrogen of 11 mg/dL, a glomerular filtration rate of >60 mL/min, and a bicarbonate level of 22 mEq/L. Her liver function tests showed an aspartate aminotransferase of 100 IU/L, an alanine aminotransferase of 66 IU/L, and an alkaline phosphatase of 128 IU/L. The patient tested positive for COVID-19 with rapid antigen and reverse transcription-polymerase chain reaction (RT-PCR) tests in the ED. The initial arterial blood gas (ABG) showed a pH of 7.36, pCO2 30, pO2 75, and an oxygen saturation of 94% on 6 L/min oxygen through NC. The chest X-ray revealed cardiomegaly and bilateral pulmonary opacities suggestive of COVID-19 pneumonia with an underlying ILD/pulmonary fibrosis (Figure 1).

**Figure 2:**
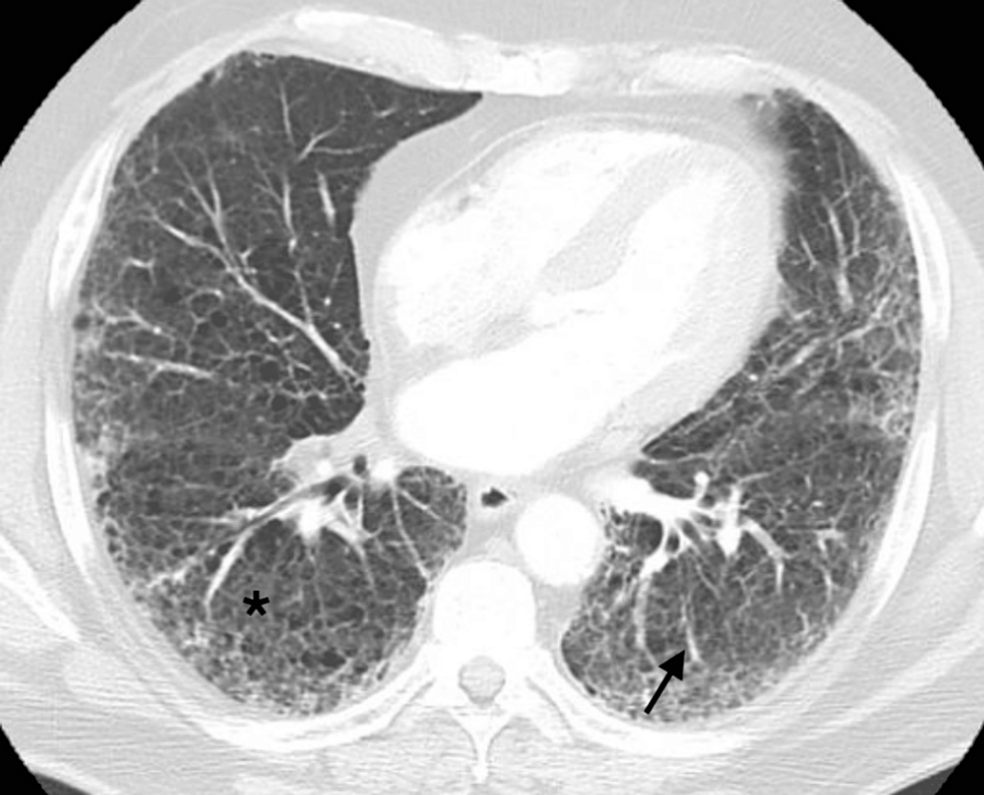
The computed tomography (CT) scan showed diffuse ground-glass alveolar airspace infiltrates (star) and underlying pulmonary fibrosis (arrow)

Further assessments included blood and respiratory cultures, and the patient was started empirically on azithromycin and ceftriaxone intravenous (IV) in the ED. She was also placed on dexamethasone IV and enoxaparin subcutaneous (SC) at a prophylactic dose. While in the ED, she became more tachypneic and started using accessory muscles for breathing. The repeated ABG showed a pH of 7.46, pCO_2_ of 25, pO_2_ of 56, and an oxygen saturation of 88% on 6 L/min oxygen through NC. She was placed on bilevel-positive airway pressure (BiPAP) and transferred to the definitive observation unit (DOU) for further evaluation and management of acute-on-chronic hypoxic respiratory failure. Over the course of 10 days, multiple subspecialties were consulted, including pulmonology, rheumatology, and infectious diseases (ID). The pulmonology service requested a computed tomography (CT) scan, which showed diffuse ground-glass alveolar airspace infiltrates and a dilated main pulmonary artery, suggesting underlying pulmonary hypertension without evidence of a pulmonary embolism (PE) (Figure 2). She never required intubation or transfer to the intensive care unit (ICU). After being on the BiPAP machine for two days in the DOU, she was transferred to the telemetry unit. On the tenth day of hospitalization, the patient was medically stable for discharge. She was advised to schedule a follow-up appointment with her primary care physician, as well as an outpatient pulmonologist and rheumatologist within one week. Additionally, she was recommended to follow up with the lung transplant team as she remained on the list for transplantation.

## Discussion

There is a crosstalk between RA and COVID-19 infection, as both share an identical mechanistic pathway of disease pathogenesis. Immune hyperactivation and several pro-inflammatory cytokines have been identified as key contributors to synovial inflammation in RA and severe lung disease in COVID-19 patients [10]. Patients with rheumatologic diseases, such as RA, have an increased risk of infection due to the immunopathogenesis of the disease itself, particularly when the disease is in its active phase, and due to being on immunosuppressive medications [11]. One study showed that elderly patients with a mean age of 65 years with rheumatic diseases, such as RA, have an increased risk of hospital admission and development of severe COVID-19 pneumonia [12]. In a large worldwide case series, it was demonstrated that patients with rheumatic diseases, notably RA, have an increased risk of death from COVID-19 infection compared to the general population [13]. Similarly, it has been reported that COVID-19 infection is associated with a higher incidence of inflammatory arthritis, especially RA, particularly in women and older patients, with the greatest increase occurring within the first year post-COVID [14]. Few studies have shown that inflammatory arthritis, including RA, can potentially be a cause of post-COVID arthritis [15,16].

Clinical management of RA can be challenging in patients infected with COVID-19 who require treatment. It has been found that using glucocorticoids ≥10 mg/day in patients with rheumatic diseases is associated with an increased risk of COVID-19 hospitalization [17]. Among 1.7 million patients with RA or osteoarthritis, it has been shown that the risk of death is lower from COVID-19 infection among users of non-steroidal anti-inflammatory drugs (NSAIDs) [18]. Among disease-modifying antirheumatic drugs (DMARDs), conventional synthetic DMARDs, except for leflunomide, methotrexate, and sulfasalazine, are considered safe to be continued in RA patients with COVID-19 infection; however, all biological DMARDs, except for interleukin 6 (IL-6) inhibitors, and all targeted synthetic DMARDs have been advised to be discontinued in patients with suspected or confirmed COVID-19 infection [10].

Vaccines are among the greatest medical accomplishments and remain a cornerstone of the COVID-19 pandemic response for preventing hospitalizations, improving long-term health outcomes, and lowering the risk of death. It has been shown that full vaccination history reduces mortality rates, disease severity, and the length of hospital stay in COVID-19-infected patients compared to those who are unvaccinated or only partially vaccinated [19]. Not being vaccinated for COVID-19 has been significantly associated with higher rates of ICU admission, mechanical ventilation, increased hospital stay durations, and death according to a prospective study [20]. In a cohort study, it was shown that full vaccination status was associated with lower mortality rates in patients who were intubated due to COVID-19-related acute respiratory distress syndrome (ARDS) [9]. In critically ill COVID-19 patients, it was found that vaccinated patients required significantly less non-invasive ventilation, had fewer intubation days, and experienced less hypoxia compared to non-vaccinated patients, as shown in a retrospective observation study [7]. Furthermore, slow vaccination and booster administration for COVID-19 were associated with higher mortality rates in “slower” European countries as compared to more rapidly immunized countries throughout all periods of the pandemic [21]. Another study recommended vaccination for COVID-19 in patients with comorbid pulmonary diseases, as they are susceptible to more severe COVID-19 with higher complications and mortality rates [22]. Patients with ILD, especially RA-ILD and chronic hypersensitivity pneumonitis, are at increased risk of death from COVID-19, particularly those with obesity and poor respiratory function parameters [23].

We presented a COVID-19-infected patient with RA-ILD who was a lung transplant candidate on long-term nintedanib for interstitial fibrosis. She was fully vaccinated and survived the acute phase of hypoxic respiratory failure without needing intubation or transfer to the ICU. This case underscores the importance of full vaccination for COVID-19 in RA patients, especially those with RA-ILD. We also recommend implementing more effective policies and public education both nationwide and globally to encourage vaccination and raise public awareness.

## Conclusions

Rheumatoid arthritis is a multisystemic autoimmune disease that involves the peripheral joints and exhibits various extra-articular manifestations. Lung involvement is a common extra-articular manifestation of RA. Patients with RA are at a greater risk of contracting COVID-19, experiencing severe symptoms, and having higher morbidity and mortality rates. The combined treatment of these two diseases poses many challenges due to immunosuppression, the presence of comorbidities, and the risk of medication interactions. The most important factor to reduce the mortality rate, notably in high-risk patients, is being fully vaccinated against COVID-19. Additionally, it is crucial to consider other potential factors, such as a patient's overall health status, co-morbidities, medication compliance, and timely medical intervention, in order to determine the overall health outcome.

## References

[1] Cimmino MA, Salvarani C, Macchioni P (2000). Extra-articular manifestations in 587 Italian patients with rheumatoid arthritis. Rheumatol Int.

[2] Yunt ZX, Solomon JJ (2015). Lung disease in rheumatoid arthritis. Rheum Dis Clin North Am.

[3] Kelly C (2022). Increased risk of severe COVID-19 outcomes in patients with rheumatoid arthritis and interstitial lung disease. Lancet Rheumatol.

[4] Favalli EG, Maioli G, Biggioggero M, Caporali R (2021). Clinical management of patients with rheumatoid arthritis during the COVID-19 pandemic. Expert Rev Clin Immunol.

[5] Zaccardelli A, Wallace ZS, Sparks JA (2023). Acute and postacute COVID-19 outcomes for patients with rheumatoid arthritis: lessons learned and emerging directions 3 years into the pandemic. Curr Opin Rheumatol.

[6] Davis MG, Akhlaq A, Aamer S, Shuja H, Edigin E, Sheikh AB (2024). COVID-19 infection and clinical outcomes in hospitalized patients with rheumatoid arthritis: insights from the National Inpatient Sample. J Community Hosp Intern Med Perspect.

[7] AlQahtani SY, Alabdulqader AA, Al Mashhour WA (2023). Clinical characteristics and outcomes of vaccinated vs non-vaccinated critically ill COVID-19 patients: retrospective observation study. Infect Drug Resist.

[8] Havaldar AA, Selvam S (2024). Estimation of the effect of vaccination in critically ill COVID-19 patients, analysis using propensity score matching. Ann Intensive Care.

[9] Grapsa E, Adamos G, Andrianopoulos I (2022). Association between vaccination status and mortality among intubated patients with COVID-19-related acute respiratory distress syndrome. JAMA Netw Open.

[10] Dewanjee S, Kandimalla R, Kalra RS (2021). COVID-19 and rheumatoid arthritis crosstalk: emerging association, therapeutic options and challenges. Cells.

[11] Akiyama S, Hamdeh S, Micic D, Sakuraba A (2021). Prevalence and clinical outcomes of COVID-19 in patients with autoimmune diseases: a systematic review and meta-analysis. Ann Rheum Dis.

[12] Alzahrani ZA, Alghamdi KA, Almaqati AS (2021). Clinical characteristics and outcome of COVID-19 in patients with rheumatic diseases. Rheumatol Int.

[13] Hyrich KL, Machado PM (2021). Rheumatic disease and COVID-19: epidemiology and outcomes. Nat Rev Rheumatol.

[14] Joo YB, Lim YH, Kim KJ, Park KS, Park YJ (2019). Respiratory viral infections and the risk of rheumatoid arthritis. Arthritis Res Ther.

[15] Yadav S, Bonnes SL, Gilman EA, Mueller MR, Collins NM, Hurt RT, Ganesh R (2023). Inflammatory arthritis after COVID-19: a case series. Am J Case Rep.

[16] Derksen VF, Kissel T, Lamers-Karnebeek FB (2021). Onset of rheumatoid arthritis after COVID-19: coincidence or connected?. Ann Rheum Dis.

[17] Gianfrancesco M, Hyrich KL, Al-Adely S (2020). Characteristics associated with hospitalisation for COVID-19 in people with rheumatic disease: data from the COVID-19 Global Rheumatology Alliance physician-reported registry. Ann Rheum Dis.

[18] Kushner P, McCarberg BH, Grange L (2022). The use of non-steroidal anti-inflammatory drugs (NSAIDs) in COVID-19. NPJ Prim Care Respir Med.

[19] Sezen YI, Senoglu S, Karabela SN (2022). Risk factors and the impact of vaccination on mortality in COVID-19 patients. Bratisl Lek Listy.

[20] Elamin MY, Maslamani YA, Alsheikh FA (2024). Impact of vaccination on morbidity and mortality in adults hospitalized with COVID-19 during the omicron wave in the Jazan Region, Saudi Arabia. Saudi Med J.

[21] Matveeva O, Shabalina SA (2023). Comparison of vaccination and booster rates and their impact on excess mortality during the COVID-19 pandemic in European countries. Front Immunol.

[22] Gülsen A, König IR, Jappe U, Drömann D (2021). Effect of comorbid pulmonary disease on the severity of COVID‐19: a systematic review and meta‐analysis. Respirology.

[23] Drake TM, Docherty AB, Harrison EM (2020). Outcome of hospitalization for COVID-19 in patients with interstitial lung disease. An international multicenter study. Am J Respir Crit Care Med.

